# Site specific incidence rate of genomic subtypes of enteropathogenic *Escherichia coli* and association with enteric inflammation and child growth

**DOI:** 10.1038/s41598-022-09730-8

**Published:** 2022-04-06

**Authors:** Rina Das, Parag Palit, Md. Ahshanul Haque, Mustafa Mahfuz, A. S. G. Faruque, Tahmeed Ahmed

**Affiliations:** Emeritus Scientist, Nutrition and Clinical Services Division, icddr,b, 68 Shaheed Tajuddin Ahmed Sharani, Dhaka, 1212 Bangladesh

**Keywords:** Microbiology, Biomarkers, Medical research, Risk factors

## Abstract

There is a lack of information highlighting the possible association between the genomic subtypes of enteropathogenic *Escherichia coli* (EPEC) on environmental enteric dysfunction (EED) and on linear growth during childhood. Genomic subtypes of EPEC from stool samples collected from 1705 children enrolled in the MAL-ED birth cohort were detected by TaqMan Array Cards. We measured site-specific incidence rate by using Poisson regression models, identified the risk factors and estimated the association of genomic subtypes of EPEC with the composite EED score and linear growth at 24 months of age. In general, the highest incidence rate (39%) was found among children having aEPEC infection, which was the greatest in Tanzania (54%). Exclusive breastfeeding and having an improved sanitation facility were found to be protective factors against EPEC infection. In the multivariate models, in overall effect after adjusting for the potential covariates aEPEC showed strong positive associations with the EED scores and tEPEC showed a positive association with poor linear growth at 24 months of age. Our analyses may lay the cornerstone for a prospective epidemiologic investigation for a potential vaccine development aimed at reducing the burden of EPEC infections and combat childhood malnutrition.

## Introduction

Diarrhea is responsible for the death of approximately 900,000 children per year worldwide, with the majority of cases of mortality occurring in developing countries^1^. Host factors such as: immunocompromisation, enteropathy and dysbiosis of the gut microbiome, which are commonly attributing to childhood malnutrition may in turn predispose malnourished young children to greater severity of diarrheal and other enteric diseases and their long-term sequelae^2,3^. Diarrheal diseases in combination with malnutrition have also been reported to hinder the cognitive development of millions of young children worldwide, with persistent infections occurring in their most formative early childhood when critical brain synaptogenesis occurs^4^.

Findings from the Global Enteric Multicenter Study (GEMS) report enteropathogenic *Escherichia coli* (EPEC) to be the leading contributor of diarrheal mortality among children in low and middle income countries (LMICs) aged less than 12 months^5^. EPEC does not possess the technology for the production of heat-labile or heat-stable enterotoxin and is classified based on a lesion known as the attaching-and-effacing (A/E) phenotype to the EPEC adherence factor (EAF) plasmid due to the *eae* gene located on the chromosomal pathogenicity island called the locus of enterocyte effacement (LEE locus) and produces the intimin adhesin^6^. EPEC is equipped with the locus of enterocyte effacement (LEE) pathogenicity island, which comprises of 41 open reading frames (ORFs) and codes for a distinct type III secretion system that is involved in the pathogenesis of these organisms^7^.

On the basis of the presence of the EAF plasmid encoding bundle-forming pili (BFP), EPEC can be sub-grouped into typical EPEC (tEPEC) and atypical EPEC (aEPEC)^8^. Typical EPEC is usually linked to incidences of gastroenteritis, even severe diarrhea among infants, while atypical EPEC is associated with a wide array of clinical manifestations, ranging from asymptomatic colonization to prolonged diarrhea, based on different settings^8–10^. Findings from studies carried out across 13 developing countries showed that isolates of aEPEC accounted for 78% of all EPEC associated diarrheal cases among children aged less than 5 years^11^. Additionally, EPEC may lead to severe nutrient malabsorption, resulting in nutritional consequences and eventual persistence of diarrhea^12^.

Results from a cross-sectional case–control study conducted among Brazilian aged between 2 and 36 months reported a higher association of tEPEC infections with clinical severity of diarrhea and undernutrition compared to aEPEC infections^13^. Later on, a recent study conducted in Bangladesh reported higher detection rates of both tEPEC and aEPEC among severely malnourished infants with diarrhea aged less than 6 months, compared to their age-matched well-nourished counterparts, although the detection of tEPEC was greater than the detection of aEPEC in both the severely malnourished and well-nourished groups^14^. Moreover, a reanalysis of the data from the Global Enteric Multicenter Study reported that tEPEC was significantly associated with higher incidences of moderate-to-severe diarrhea (MSD) among children aged 6–11 months and suffering from acute malnutrition^15^.

However, a thorough understanding of the epidemiology of infection by the EPEC subtypes is yet to be established, owing to the lack of proper discrimination in many studies^16^. The main goal of our study was to estimate the site-specific incidence rates of the two genomic subtypes of EPEC (tEPEC and aEPEC) and their possible associations with the composite EED (environmental enteric dysfunction) scores and the consequent growth failure among children at 24 months of age.

## Results

### General characteristics of the study population and incidence rates of virulence-related genes associated with EPEC

A total of 34,622 monthly stool samples were collected from 1715 participants who completed the follow-up to 24 months. All the stool samples collected over this time from all the participants at the different study sites were assessed for the presence of virulence-related genes associated with EPEC using TaqMan Array Cards (TAC). The general characteristics of the study children are presented in Table 1.

**Table 1:** General characteristics of the MAL-ED study children (n = 1715) from Bangladesh, India, Nepal, Pakistan, South Africa, Tanzania, Brazil, and Peru from November 2009 to February 2012.

Characteristics n (%)	Bangladesh	Brazil	India	Nepal	Peru	Pakistan	South Africa	Tanzania	Overall
Gender (male)	108 (51.43)	89 (53.94)	105 (46.26)	122 (53.74)	105 (54.12)	120 (48.78)	120 (50.63)	105 (50.24)	874 (50.96)
Days of exclusive breastfeeding^c^	143.2 ± 42.7	93.7 ± 57.8	105.4 ± 42.9	92.5 ± 54.5	89.5 ± 61.3	19.9 ± 22.7	38.6 ± 26.3	62.2 ± 35	78.6 ± 57.7
Birth weight (Kg)^c^	2.8 ± 0.4	3.4 ± 0.5	2.9 ± 0.4	3.0 ± 0.4	3.1 ± 0.4	2.7 ± 0.4	3.2 ± 0.5	3.2 ± 0.5	3.0 ± 0.5
Weight for age z score at enrollment^c^	− 1.3 ± 0.9	− 0.2 ± 1.0	− 1.3 ± 1.0	− 0.9 ± 1.0	− 0.6 ± 0.9	− 1.4 ± 1.0	− 0.4 ± 1.0	− 0.1 ± 1.1	− 0.8 ± 1.1
Length for age z score at enrollment^c^	− 1.0 ± 1.0	− 0.8 ± 1.1	− 1.0 ± 1.1	− 0.7 ± 1.0	− 0.9 ± 1.0	− 1.3 ± 1.1	− 0.7 ± 1.0	− 1.0 ± 1.1	− 0.9 ± 1.1
Length for age z score at 24 months^c^	− 2.0 ± 0.9	0.0 ± 1.1	− 1.9 ± 1.0	− 1.3 ± 0.9	− 1.9 ± 0.9	–	− 1.7 ± 1.1	− 2.7 ± 1.0	− 1.7 ± 1.2
Weight for length z score at 24 months^c^	− 0.8 ± 0.9	0.5 ± 1.4	− 0.9 ± 0.9	− 0.3 ± 0.9	0.3 ± 0.9	–	0.5 ± 1.0	0.1 ± 1.0	− 0.1 ± 1.1
Maternal age (months)^c^	25 ± 5	25.4 ± 5.6	23.9 ± 4.2	26.6 ± 3.7	24.8 ± 6.3	28.1 ± 5.9	27 ± 7.2	29.1 ± 6.5	26.3 ± 5.9
Maternal BMI^ac^	22.3 ± 3.4	25.7 ± 4.4	22.0 ± 4.0	25.1 ± 3.2	24.9 ± 3.7	21.5 ± 3.8	27 ± 5.5	22.9 ± 3.2	23.9 ± 4.4
Maternal education level (< 6y)	133 (63.3)	22 (13.3)	80 (35.2)	59 (26)	44 (22.7)	202 (82.1)	5 (2.1)	75 (35.9)	620 (36.2)
Mother has < 3 living children	160 (76.2)	113 (68.5)	157 (69.8)	199 (87.7)	111 (57.2)	105 (42.7)	141 (59.5)	58 (27.8)	1044 (61)
Ownership of cattle	1 (0.5)	0	5 (2.2)	3 (1.3)	0	146 (59.4)	33 (13.9)	157 (75.1)	345 (20.1)
Ownership of chicken	3 (1.4)	1 (0.6)	14 (6.2)	73 (32.2)	75 (38.7)	144 (62.3)	87 (37.2)	204 (97.6)	601 (35.4)
Routine treatment of drinking water	130 (61.9)	10 (6.1)	7 (3.1)	98 (43.2)	32 (16.5)	0	12 (5.1)	12 (5.7)	301 (17.6)
Improved drinking water source	210 (100)	165 (100)	227 (100)	227 (100)	184 (94.9)	246 (100)	196 (82.7)	89 (42.6)	1544 (90.0)
Improved floor	204 (97.1)	165 (100)	222 (97.8)	109 (48)	69 (35.6)	81 (32.9)	231 (97.5)	13 (6.2)	1094 (63.8)
Improved sanitary latrine	210 (100)	165 (100)	121 (53.3)	227 (100)	66 (34)	197 (80.1)	232 (97.9)	19 (9.1)	1237 (72.1)
Monthly income < 150 USD	69 (32.9)	161 (97.6)	19 (8.4)	106 (46.7)	58 (29.9)	115 (46.8)	179 (75.5)	0	707 (41.2)
< 2 people live in per room	8 (3.8)	141 (85.5)	46 (20.3)	126 (55.5)	122 (62.9)	27 (10.9)	201 (84.8)	95 (45.5)	766 (44.7)
Average serum zinc level (mmol/l)^db^	11.3 (10.6, 12.1)	14 (13, 14.9)	9.1 (8.6, 9.6)	11.2 (10.4, 12.2)	14.8 (13.1, 17.9)	8.9 (7.7, 10)	22.9 (14.3, 32.9)	11.1 (9.9, 12.3)	11.3 (9.6, 13.7)
Average AGP (mg/dL)^bd^	84.3 (71.5,105.3)	95.7 (81, 117)	97 (83, 110)	117.7 (102.7, 139)	115 (98, 130.3)	93 (77.5, 111.8)	126 (107.3, 153, 7)	114.3 (97.7, 138.7)	106.3 (87, 127)

^a^BMI: body mass index.

^b^Average of 7, 15 and 24-months serum zinc and plasma AGP (alpha-1-acid glycoprotein) level.

^c^Mean ± Standard deviation.

^d^Median (IQR).

**Figure 1:**
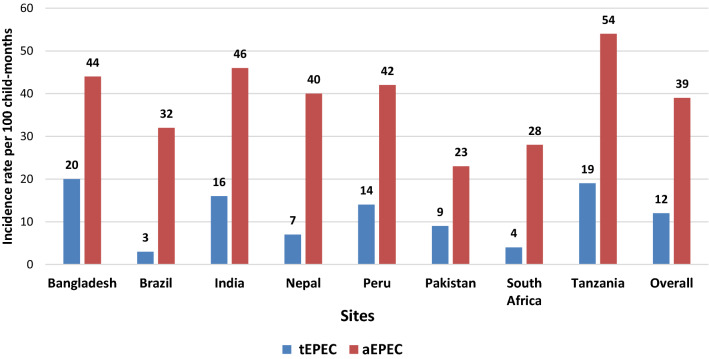
The incidence rate of infection with the two genomic subtypes of EPEC (tEPEC and aEPEC) across each of the eight study sites (Bangladesh, India, Nepal, Pakistan, South Africa, Tanzania, Brazil, and Peru) from November 2009 to February 2012.

The incidence rates of the two genomic subtypes of EPEC (tEPEC and aEPEC) in the stool samples collected across all the 8 study sites over the 24 months study period have been shown in Fig. 1. The overall incidence rate of the aEPEC was the highest (39%). The incidence of virulence-related genes associated with aEPEC was highest in Tanzania (54%). It was also observed that the overall incidence of tEPEC virulence gene was lowest among all the sites (3%). The incidence of aEPEC infection was higher than the incidence of tEPEC infection, across all the study sites (Supplementary Table 1).

### Factors associated with the two genomic subtypes of EPEC

Factors associated with infections by the two genomic subtypes of EPEC across all study sites were identified using Poisson regression (Table 2). The incidence rate for infection of EPEC in female children was comparable with male children. Additionally, exclusive breastfeeding status with infections by the aEPEC [IRR:0.99 (95%CI: 0.99–1.00); *p* < 0.001] and improved sanitation among children infected with tEPEC [IRR: 0.90 (95%CI: 0.81–0.99); *p* = 0.026] were associated and found to be statistically significant. The incidence rate of aEPEC was higher for the sites of Brazil, Nepal, South Africa, and Peru, while that of the aEPEC was the greatest in Peru. Consequently, the incidence rate of tEPEC infection was the lowest in Brazil and that of aEPEC was the lowest in Pakistan. The incidence rate of tEPEC infection was higher in India and Peru (Table 2).

**Table 2:** Factors associated with infections by the two genomic subtypes of EPEC across each of the eight study sites (Bangladesh, India, Nepal, Pakistan, South Africa, Tanzania, Brazil, and Peru) from November 2009 to February 2012.

Factors	tEPEC		aEPEC	
	IRR (95% CI)	*P* value	IRR (95%CI)	*P* value
**Sex**				
Male	1.06 (0.99, 1.13)	0.054	1.00 (0.96, 1.03)	1.00
Exclusive BF (months)	1.00 (0.99, 1.00)	0.901	0.99 (0.99, 1.00)	< 0.001
**Cattle**				
Yes	0.90 (0.80, 1.02)	0.096	0.94 (0.88, 1.01)	0.086
**Chickens**				
Yes	1.02 (0.93, 1.13)	0.647	1.03 (0.98, 1.08)	0.247
WAZ	0.99 (0.96, 1.03)	0.706	1.01 (0.99, 1.03)	0.313
**Improved floor**				
Yes	0.93 (0.84, 1.03)	0.175	0.99 (0.94, 1.05)	0.706
**Improved drinking water source**				
Yes	0.98 (0.86, 1.11)	0.699	1.00 (0.93, 1.07)	0.943
**Use water treatment method**				
Yes	0.96 (0.87, 1.06)	0.418	1.00 (0.95, 1.05)	0.972
**Improved sanitation**				
Yes	0.90 (0.81, 0.99)	0.026	0.98 (0.93, 1.04)	0.459
Maternal Age	0.99 (0.99, 1.00)	0.059	1.00 (0.99, 1.00)	0.295
**Mother has < 3 living children**				
Yes	0.93 (0.86, 1.01)	0.103	0.96 (0.92, 1.01)	0.119
**< 2 people live in per room**				
Yes	0.99 (0.91, 1.07)	0.75	0.98 (0.94, 1.02)	0.356
**Monthly income < 150 USD**				
Yes	1.05 (0.96, 1.14)	0.287	1.00 (0.95, 1.05)	0.998
Maternal education (years)	0.99 (0.98, 1.00)	0.054	1.00 (0.99, 1.00)	0.162
**Country**				
Brazil	0.17 (0.14, 0.22)	< 0.001	0.71 (0.65, 0.79)	< 0.001
India	0.76 (0.67, 0.87)	< 0.001	0.99 (0.92, 1.07)	0.839
Nepal	0.34 (0.29, 0.40)	< 0.001	0.87 (0.81, 0.94)	0.001
Peru	0.62 (0.53, 0.73)	< 0.001	0.88 (0.81, 0.97)	0.010
Pakistan	0.44 (0.37, 0.53)	< 0.001	0.48 (0.43, 0.53)	< 0.001
South Africa	0.23 (0.19, 0.28)	< 0.001	0.60 (0.54, 0.66)	< 0.001
Tanzania	0.82 (0.66, 1.02)	0.070	1.13 (1.00, 1.27)	0.057

Model: Poisson regression; IRR: Incidence rate ratio; aWAZ: Weight-for-age z score.

Dependent variable: Number of infections during follow up (1–24 m).

Offset: Log of the total number of follow up; alpha (α): dispersion parameter.

Adjusted for the site and all variables included in the multivariable model.

### Association between the different virulence genes of EPEC and child growth

Infections with tEPEC were associated with poor linear growth (difference in 24 months LAZ: length-for-age z score), with a stronger association being observed for overall all the study sites (Table 3). In Nepal, infection with tEPEC [− 2.44 difference in 24 months LAZ (95% CI: − 4.36, −0.52); *p* = 0.013] had a statistically significant negative association with LAZ. In India, aEPEC [− 0.96 difference in 24 months LAZ (95% CI: − 1.87, − 0.05); *p* = 0.040] was negatively associated with LAZ which was also statistically significant. For the other countries (except Bangladesh) infections with tEPEC were negatively associated with LAZ and for the other three countries (Tanzania, Brazil, South Africa) infections with aEPEC were negatively associated with LAZ but were not statistically significant.

**Table 3:** Genomic subtypes of EPEC and burden on child growth at 24 months across each of the study sites (except Pakistan) from November 2009 to February 2012.

Site	Length-for-age z score			
	tEPEC		aEPEC	
	Coef. (95% CI)	*P* value	Coef. (95% CI)	*P* value
Bangladesh	0.39 (− 0.82, 1.61)	0.522	0.02 (− 0.91, 0.94)	0.973
Brazil	− 1.32 (− 4.74, 2.10)	0.447	− 0.32 (− 1.61, 0.98)	0.630
India	− 0.66 (− 2.09, 0.78)	0.368	− **0.96 (**− **1.87, **− **0.05)**	**0.040**
Nepal	− **2.44 (**− **4.36, **− **0.52)**	**0.013**	0.47 (− 0.42, 1.36)	0.304
Peru	− 0.50 (− 2.10, 1.09)	0.535	0.41 (− 0.61, 1.42)	0.431
South Africa	− 2.79 (− 6.05, 0.48)	0.094	− 0.60 (− 1.78, 0.58)	0.320
Tanzania	− 0.81 (− 3.02, 1.41)	0.471	− 0.55 (− 2.39, 1.29)	0.555
Overall	− **0.89 (**− **1.57, **− **0.21)**	**0.010**	− 0.29 (− 0.69, 0.11)	0.151

Adjusted in the linear regression model for sex, birth weight, exclusive breastfeeding, WAMI Index (water/sanitation, assets, maternal education, and income); enrollment length-for-age z score; maternal BMI; poultry and cattle in house, a mother has less than 3 living children, average serum zinc level, average AGP (alpha-1-acid glycoprotein) level, presence of co-pathogens (*Campylobacter,* LT-ETEC, ST-ETEC, *Shigella/*EIEC, and *Giardia)* and site for overall estimate.

Coef. Coefficient, CI confidence interval.

### Association between genomic subtypes of EPEC and enteric inflammation

After adjusting for the potential covariates like age, sex, WAMI index (water/sanitation, assets, maternal education, and income); enrollment length-for-age z score; maternal BMI; the number of children in the household, presence of poultry/cattle in the household, seasonality, serum zinc level, AGP (alpha-1-acid glycoprotein), presence of co-pathogens (*Campylobacter*, LT-ETEC, ST-ETEC, *Shigella*/EIEC, and *Giardia*), site for the overall estimate and age as the time variable in GEE (generalized estimating equations) model for the genomic subtypes of EPEC, aEPEC was also clearly and consistently associated with increased EED score with the overall [Coef. 0.15 (95% CI: 0.07, 0.23); *p* < 0.001] effect for all sites. The same findings were observed in India [Coef. 0.19 (95% CI: 0.01, 0.37); *p* = 0.043] and Nepal [Coef. 0.31 (95% CI: 0.11, 0.50); *p* = 0.002] individually for aEPEC infection (Table 4).

**Table 4:** Association between the genomic subtypes of EPEC and enteric inflammation (EED score: stool Myeloperoxidase, Neopterin, and Alpha-1-Antitrypsin).

Site	EED score			
	tEPEC		aEPEC	
	Coef. (95% CI)	*P* value	Coef. (95% CI)	*P* value
Bangladesh	− 0.09 (− 0.35, 0.17)	0.506	0.10 (− 0.11, 0.31)	0.338
Brazil	− 0.20 (− 0.93, 0.54)	0.601	0.21 (− 0.08, 0.51)	0.160
India	0.04 (− 0.20, 0.28)	0.729	0.19 (0.01, 0.37)	0.043
Nepal	0.11 (− 0.25, 0.47)	0.552	0.31 (0.11, 0.50)	0.002
Peru	0.27 (− 0.08, 0.63)	0.134	0.23 (− 0.02, 0.48)	0.073
Pakistan	− 0.03 (− 0.33, 0.27)	0.848	0.001 (− 0.22, 0.22)	0.975
South Africa	0.31 (− 0.27, 0.89)	0.290	0.02 (− 0.23, 0.28)	0.860
Tanzania	0.19 (− 0.09, 0.47)	0.187	0.20 (− 0.02, 0.43)	0.081
Overall	0.05 (− 0.07, 0.16)	0.427	0.15 (0.07, 0.23)	< 0.001

The dependent variable was log (MPO, NEO, and ALA); Independent variables: the presence of virulence-related genes associated with EPEC at each month.

Adjusted in GEE model for sex, age, WAMI Index (water/sanitation, assets, maternal education, and income); enrollment length-for-age z score; maternal BMI; the number of children, poultry/cattle in house, seasonality, serum zinc level, AGP (alpha-1-acid glycoprotein), presence of co-pathogens (*Campylobacter,* LT-ETEC, ST-ETEC, *Shigella,* and *Giardia*), site for the overall estimate, and age as the time variable. EED score (*AAT* alpha-1-anti-trypsin, *MPO* myeloperoxidase, *NEO* neopterin).

Coef. Coefficient, CI confidence interval.

## Discussion

To our knowledge, this is the first study investigating the associations between the two prominent genomic subtypes of EPEC (tEPEC and aEPEC) with enteric inflammation and linear growth in children from birth up until 2 years of age. Several EPEC virulence genes have been used in case–control and epidemiological studies in recent years, with *eae* and *bfpA* being among the most common for detection of the prominent subtypes of EPEC^17^.

We found that exclusive breastfeeding and having an improved sanitation facility had a very small protective effect against both tEPEC and aEPEC. Concurrent observation from a study conducted among Tanzanian children have described the same findings, most of the children younger than 6 months were exclusively breastfeed whose stool specimens were negative for EPEC^18^. Another study conducted in University Hospital, Sao Paulo, Brazil over 46 women who had given birth to normal term babies found that, the human colostrum IgA antibodies reacting to enteropathogenic *E. coli* antigens and their persistence in the gastrointestinal tract was shown by the strong reactivity to the 94-kDa band in the Western blot analysis. These data confirm the role of colostrum antibodies in protecting the neonate against infections caused by EPEC^19^. Hands, soil, and water could all be key sources of exposure to EPEC. The most important reservoirs of pathogens are the ones that children come in contact with most often. Overall, the sanitation intervention seems to have a limited effect on the presence of EPEC in hands, soil, and water^20^. Such assessments of different factors that may be associated with EPEC infections may in turn enhance in devising new treatment strategies against EPEC infections.

We documented high incidence rates of both tEPEC and aEPEC in Tanzania. The Global Enteric Multicenter Study (GEMS) determined that the diarrheal death of children can largely be attributed to a mere few infectious agents^5^. In particular, diarrhea caused by tEPEC is associated with a 2.6-fold higher hazard of death, the largest reported in the GEMS^5^. In our study, the lowest incidence rate of tEPEC has been found in Brazil. Other studies reported aEPEC to have a significant association with diarrhea in several countries, including Brazil^21–23^, where aEPEC was more prevalent than tEPEC. These observations indicate that aEPEC infections have significant clinical pertinence regarding the burden of EPEC infections and demand greater vigilance through epidemiologic and virulence studies.

In diarrheal animal models, positive culture or qPCR results for atypical EPEC (aEPEC) had significantly higher small intestinal and colonic lesion scores than a healthy animal. The increase in colonic lesion scores in animals with diarrhea and aEPEC infections was due to increased amounts of inflammatory infiltrate in the lamina propria^24^. In our study, the presence of aEPEC was more strongly associated with EED scores, implying a higher intestinal inflammation. The relevance of elevated intestinal inflammation associated with this particular genomic subtype of EPEC is not yet clear and no evidence indicates a particular genomic subgroup of EPEC to be associated with elevated intestinal inflammatory biomarkers and subsequently increased EED score. Henceforth, our study is the first attempt undertaken towards the generation of evidence-based knowledge of the contribution of the different genomic subgroups of EPEC with regards to enteric inflammation and poor child growth in LMIC settings.

Our study findings also illustrate that the presence of tEPEC and aEPEC was negatively associated with childhood linear growth in Nepal and India, respectively. However, in Norwegian children a significant association was observed with diarrhea lasting 14 days or more, a finding that may indicate a role for atypical EPEC in prolonged diarrhoeal episode^25^ and which might cause chronic malnutrition. When the different genomic subgroups of EPEC adhere to epithelial cells in vitro or in vivo they cause characteristic changes known as Attaching and Effacement (A/E) lesions. Decrease in number and height of microvilli, blunting of borders of enterocytes, loss of the glycocalyx, shortening of villi, and presence of a mucus pseudo membrane coating the mucosal surface were the abnormalities observed in the majority of children^12^. These ultrastructural derangements may be due to a mechanism where the different subgroups of EPEC trigger diarrhea as a response to contamination of food or water, which in turn if persistent may, in turn, result in chronic malnutrition in an early phase of life.

Although antibiotic therapy is not recommended for cases of mild acute diarrhea^26^, studies on the determination of the antibiotic resistance profile of aEPEC are necessary, due to cases of persistent diarrhea associated with aEPEC being reported^25–27^. Henceforth, under such clinical manifestations, the administration of antimicrobial therapy may be useful^28^.

Despite some potentially promising nature of our study findings, there are several possible limitations associated with our analysis. As an observational cohort study, the causality of the associations between infection with various genomic subgroups of EPEC and both intestinal inflammation and linear growth cannot be proven but can be hypothesized based on several factors, including the appropriate adjustment of the models for possible confounders, the strength and consistency of the associations, and the biological plausibility. We were unable to establish a temporal relationship between infections and the outcomes, which would require structured longitudinal models. Moreover, our analysis lacked data of the host gut microbiome and host diet, which have been found to be linked with infection by a wide range of enteropathogens.

The present study concludes that exclusive breastfeeding and improved sanitation facility are the possible protective factors for EPEC infections by different virulence genes and thereby leading to a compromised linear growth in childhood. The burden of both tEPEC and aEPEC was associated with increased enteric inflammation among children in the first 2 years of life.

## Method

### Study design and participants

MAL-ED (Etiology, Risk Factors, and Interactions of Enteric Infections and Malnutrition and the Consequences for Child Health) was a birth cohort study performed across 8 sites in South America, sub-Saharan Africa, and South Asia. The MAL-ED study design and methodology have been described elsewhere^29^. Briefly, 1715 children were enrolled from November 2009 to February 2012 from the community within 17 days of birth across eight different sites, namely: Bangladesh, India, Nepal, Pakistan, South Africa, Tanzania, Brazil, and Peru. In our current analysis, data from all 1715 participants were available from enrolment soon after birth up to 24 months of age.

### Ethical consideration

The study was approved by the ethical committees at each of the participating institutes across each of the 8 study sites^29^. The study was approved by the Research Review Committee and the Ethical Review Committee, icddr,b (BGD); Committee for Ethics in Research, Universidade Federal do Ceara; National Ethical Research Committee, Health Ministry, Council of National Health (BRF); Institutional Review Board, Christian Medical College, Vellore; Health Ministry Screening Committee, Indian Council of Medical Research (INV); Institutional Review Board, Institute of Medicine, Tribhuvan University; Ethical Review Board, Nepal Health Research Council; Institutional Review Board, Walter Reed Army Institute of Research (NEB); Institutional Review Board, Johns Hopkins University; PRISMA Ethics Committee; Health Ministry, Loreto (PEL); Ethical Review Committee, Aga Khan University (PKN); Health, Safety and Research Ethics Committee, University of Venda; Department of Health and Social Development, Limpopo Provincial Government (SAV); Medical Research Coordinating Committee, National Institute for Medical Research; Chief Medical Officer, Ministry of Health and Social Welfare (TZH)^30^. Written informed consent was obtained from the parents or legal guardians of every child.

All 8 of the MAL-ED sites contributed their expertise, explained the challenges unique to their site, debated vigorously, compromised, and created 1 set of consensus standard operating procedures (SOPs), which was then implemented at the sites prior to study recruitment. All methods and procedures that have been used in this study were performed in accordance with the relevant guidelines and regulations^31–34^.

### Data collection

Anthropometric measurements were taken at monthly intervals up to the age of 24 months using standard scales (Seca gmbh & co. kg., Hamburg, Germany). Length-for-age z score (LAZ), weight-for-age z score (WAZ), and weight-for-length z score (WLZ) were calculated through the use of the 2006 WHO standards for children^35^. Details of illness and child feeding practices were collected during household visits, conducted twice weekly^32^. Additionally, household demographics, presence of siblings, maternal characteristics, and other data on the child’s birth and anthropometry were obtained at enrollment^29^. Beginning at 6 months of age, socioeconomic data were collected every 6 months. The WAMI score (Water, sanitation, hygiene, Asset, Maternal education, and Income index, ranging from 0 to 1) is a socioeconomic status index. It includes access to improved water and sanitation, eight selected assets, maternal education, and household income as a representative of the socioeconomic status of the households^36^. A better socioeconomic status is indicated by a higher WAMI score^37^. Improved water and sanitation were defined following World Health Organization guidelines^38^. Treatment of drinking water was defined as filtering, boiling, or adding bleach^39^.

### Collection of stool and blood samples

Non-diarrheal stool samples were collected monthly (at least 3 days before or after a diarrheal episode) from birth to age 2 years and peripheral blood was collected at 7, 15, and 24 months of age^40^. Raw stool aliquots and blood samples were processed at all sites using harmonized protocols and stored at − 80 °C freezers before subsequent laboratory analyzes^31^.

In this study, plasma zinc was assessed as the measure of zinc status at the age of 7, 15, and 24 months. Plasma zinc concentration is a proxy marker and recommended for assessment of population zinc status, especially for children in low-income countries^41^. Plasma alpha-1-acid glycoprotein (AGP) level was considered as a biomarker for systemic inflammation and was also assessed at 7, 15, and 24 months^42^.

### Assessment of enteropathogens by TaqMan Array Cards (TAC)

Total nucleic acid (both DNA and RNA) was extracted from the fecal samples using the QIAamp Fast DNA Stool Mini kit (Qiagen), following the manufacturer’s guidelines. Two external controls, namely: MS2 bacteriophage and Phocine herpesvirus (PhHV) were added to the samples for the confirmation of nucleic acid extraction and amplification efficiency^43^.

For the detection of enteropathogens, a quantitative polymerase chain reaction (qPCR) with the use of a customized TaqMan Array Card (TAC) involving compartmentalized probe-based real-time PCR assays was used for the detection of a possible 29 pathogens from each of the samples^44^. Ct (quantification cycle) value of 35 was set as a threshold for analysis, whereby a Ct > 35 was considered as negative, as mentioned elsewhere^43^. In our current study, we investigated the occurrence of putative virulence-related genes (VRG) of EPEC, namely: *bfpA* for tEPEC, and *eae* for aEPEC.

### Assessment of biomarkers of intestinal inflammation

Intestinal inflammation was evaluated by measuring the levels of the biomarkers: alpha-1-anti-trypsin (Biovendor, Chandler, NC), neopterin (GenWay Biotech, San Diego, CA), and myeloperoxidase (Alpco, Salem, NH) in the stool samples collected from the study participants at the 3, 6, 9, 15, and 24 months of age time points by quantitative ELISA, using manufacturer’s guidelines^45^.

### Statistical analysis

All statistical analyses were performed in STATA 15 (Stata Corporation, College Station, TX). Descriptive statistics such as proportion, mean and standard deviation (SD) for symmetric data, and median with inter‐quartile range (IQR) for asymmetric quantitative variables were used to summarize the data. Chi-square and proportion test were used to determine the association between two categorical variables and t-test was used to assess the mean difference between two groups for symmetric distribution. Incidence rates were calculated using Poisson regression where outcome variables were the number of infections of EPEC (different genomic strain) and offset variables were a log of a number of follow-up visits. The factors associated with virulence-related genes associated with EPEC in the monthly stool samples were calculated using Poisson regression models. In the final multiple Poisson regression model, the following variables were considered for inclusion using stepwise forward selection: child sex, birth weight, duration of exclusive breastfeeding in months, enrollment weight for age z-score, length for age z score, maternal age in years, maternal education, mother having less than 3 living children, maternal BMI, routine treatment of drinking water, improved sanitation, household ownership of cattle/poultry, and less than 2 people live in per room. We excluded children from the Pakistan site for growth analysis, owing to bias noted in a subset of this cohort within the study period. Myeloperoxidase (MPO), neopterin (NEO), and alpha-1-antitrypsin (AAT) values were log‐transformed before the analysis. At each time point, the composite EED score ranging from 0 to 10 was calculated from the three fecal markers, as described in the previous literature by MAL-ED researchers^46,47^. Categories were assigned values as 0 (low), 1 (medium), or 2 (high). The formula for the composite EED score is as follows^48^:

$${\text{EED}}\;{\text{score}} = 2 \times {\text{AAT}}\;{\text{category}} + 2 \times {\text{MPO}}\;{\text{category}} + 1 \times {\text{NEO}}\;{\text{category}}.$$

Associations between virulence-related genes linked with EPEC and composite EED score were estimated using generalized estimating equations (GEE) to fit regression models after adjusting for sex, age, water/sanitation, assets, maternal BMI, and (WAMI) index; enrollment length-for-age and weight-for-age z score, maternal height; poultry/cattle in house, serum zinc level, inflammatory biomarker AGP (alpha-1-acid glycoprotein), presence of co-pathogens (*Campylobacter*, LT-ETEC, ST-ETEC, *Shigella*/EIEC, and *Giardia*), seasonality, and site for overall estimate and age in the month as time variable^49^. To assess and compare the associations of virulence-related genes linked with EPEC infection burden on growth at 24 months of age, we used multivariable linear regression after adjusting for the site and the necessary covariates. To detect multicollinearity, the variance inflation factor (VIF) was calculated, and no variable producing a VIF value > 5 was found in the final model. We calculated the strength of association by estimating the coefficient and its 95% CI (confidence interval) during the multivariable analysis. A *p* value of < 0.05 was considered statistically significant.

## Supplementary Information

Supplementary Table 1.

## Data Availability

A publicly available MAL-ED dataset was analyzed in this study. This data can be obtained from here: ClinEpiDB [https://clinepidb.org/ce/app/record/dataset/DS_841a9f5259].
