# Supplementary material for: Site specific incidence rate of genomic subtypes of enteropathogenic Escherichia coli and association with enteric inflammation and child growth
Source: Sci Rep. 2022 Apr 6;12:5724. doi: 10.1038/s41598-022-09730-8 (PMC8986875; doi:10.1038/s41598-022-09730-8)
Supplement: Supplementary file 1 — Supplementary Table 1. [file 41598_2022_9730_MOESM1_ESM.docx]

**Supplementary table 1.** The incidence rate of infections with the two genomic subtypes of EPEC (tEPEC and aEPEC) across each of the eight study sites (Bangladesh, India, Nepal, Pakistan, South Africa, Tanzania, Brazil, and Peru) from November 2009 to February 2012

| **Genomic subtype of EPEC** | **tEPEC** | **aEPEC** |
| --- | --- | --- |
| **Sites** | **Incidence rate (95% CI)** |  |
| Bangladesh | 0.20 (0.19-0.21) | 0.44 (0.42, 0.46) |
| Brazil | 0.03 (0.03- 0.04) | 0.32 (0.30, 0.34) |
| India | 0.16 (0.15- 0.17) | 0.46 (0.44, 0.47) |
| Nepal | 0.07 (0.06- 0.08) | 0.40 (0.38, 0.41) |
| Peru | 0.14 (0.12, 0.15) | 0.42 (0.40, 0.43) |
| Pakistan | 0.09 (0.09, 0.10) | 0.23 (0.22, 0.25) |
| South Africa | 0.04 (0.04, 0.05) | 0.28 (0.26, 0.29) |
| Tanzania | 0.19 (0.17, 0.20) | 0.54 (0.51, 0.56) |
| Overall | 0.12 (0.11, 0.12) | 0.39 (0.38, 0.39) |

95%CI: 95% confidence interval; Incidence rates were calculated using Poisson regression where outcome variables were a number of infections of EPEC (different genomic strain) and offset variables were a log of the number of follow up visits

**Data Availability**

A publicly available MAL-ED dataset was analyzed in this study. This data can be obtained from here: ClinEpiDB [htt9ps://clinepidb.org/ce/app/record/dataset/DS_841a9f5259].
